# Robotic Assistance in Simultaneous Bilateral Medial Unicompartmental Knee Arthroplasty: A Retrospective Cohort Study of 126 Knees Demonstrating Enhanced Radiographic Accuracy and Comparable Safety to Conventional Methods

**DOI:** 10.1016/j.artd.2024.101594

**Published:** 2025-01-21

**Authors:** Valentina Rossi, Constant Foissey, Andreas Fontalis, Gabriel Gaggiotti, Stefano Gaggiotti, Elvire Servien, Sébastien Lustig

**Affiliations:** a Department of Public Health, Orthopaedic Unit, Federico II University, Naples, Italy; b Department of Orthopedic Surgery and Sport Medicine, Croix-Rousse Hospital, FIFA Medical Center of Excellence, Lyon, France; c Department of Trauma and Orthopaedic Surgery, University College Hospital, London, UK; d Division of Surgery and Interventional Science, University College London, London, UK; e Orthopaedic Unit, Sanatorio Mayo, Santa Fe, Argentina; f EA 7424, Interuniversity Laboratory of Human Movement Science, Université Lyon 1, Lyon, France; g Université de Lyon, Université Claude Bernard Lyon 1, IFSTTAR, Lyon, France

**Keywords:** Unicompartmental knee arthroplasty, Bilateral, One stage procedure, Single stage, Robotic arm assistance, Conventional

## Abstract

**Background:**

One-stage bilateral unicompartmental knee arthroplasty (BUKA) is a promising option for patients with bilateral medial knee osteoarthritis. This study aims to compare the safety, early clinical and functional outcomes, and radiological results of conventional vs robotic-assisted medial BUKA.

**Methods:**

A retrospective cohort study was conducted involving patients who underwent medial BUKA as a single-stage procedure between April 2016 and January 2022. The study included both conventional (36 procedures) and robotic-assisted techniques (90 procedures) with a minimum follow-up of 6 months. Conventional procedures were performed either simultaneously by two surgical teams or sequentially by one team. Robotic procedures were exclusively performed sequentially by a single team. Data on surgical outcomes, patient-reported outcome measures (International Knee Society score), and radiographic measurements were collected.

**Results:**

Among the 63 patients analyzed, robotic-assisted procedures took significantly longer (115 ± 22 minutes) compared to conventional approaches (86.9 ± 12 minutes; *P* < .0001). No significant differences were observed in complications, length of hospital stay, rehospitalizations, patient-reported outcome measures, or overall clinical outcomes. However, radiographic analysis showed superior joint line restoration in the robotic group (−0.2 ± 0.7 mm vs −1.4 ± 1.35 mm, *P* = .03) and better tibial implant varus control (0.3° ± 0.6 vs 1° ± 1.8 degrees, *P* = .03).

**Conclusions:**

While robotic-assisted BUKA resulted in longer operative times, clinical outcomes were comparable. Radiographic findings indicated improved implant positioning, suggesting potential benefits in implantation accuracy that warrant further research.

**Level of Evidence:**

IV.

## Introduction

Among patients undergoing knee arthroplasty, 36%-70% are likely to require subsequent arthroplasty surgery on the contralateral knee within a decade [1]. The most prevalent manifestation of bilateral knee osteoarthritis is the involvement of the medial compartment in both knees [2]. In such scenarios, bilateral medial unicompartmental knee arthroplasty (BUKA) presents as an attractive treatment alternative that could be associated with less invasiveness, shorter surgical time and hospital stay, faster recovery, improved functional outcomes, and a lower incidence of complications compared to total knee arthroplasty (TKA) [[3], [4], [5], [6], [7], [8], [9], [10], [11]].

Bilateral knee arthroplasties can be performed either as a single surgical procedure or as two distinct procedures spaced over a predefined interval. A systematic review conducted in 2021 evaluated the safety and outcomes of single-stage vs two-stage BUKA, reporting no significant difference between the two approaches [12]. Further research has indicated that bilateral simultaneous unicompartmental knee arthroplasty (UKA) is not associated with an increased risk of perioperative complications or inferior outcomes. Specifically, comparable opioid consumption, pain scores, and recovery rates between bilateral simultaneous UKA and a staged approach have been reported [[13], [14], [15]]. Moreover, bilateral simultaneous UKA may result in reduced hospital costs [15].

Notably, there is paucity of evidence regarding the use of robotic assistance in the context of BUKA. Previous research has suggested that robotic-assisted UKA may enhance accuracy, safety, and functional outcomes [[16], [17], [18], [19]]. In addition, the only study published on the use of robotic assistance in the context of bilateral staged TKA has suggested its association with a reduced transfusion rate [20].

This study aimed to compare surgical and early clinical outcomes, radiological outcomes, and safety in medial BUKA performed during a single surgical session with and without robotic assistance, with a minimum follow-up period of 6 months. The hypothesis was that early clinical outcomes and complications were comparable between groups, while radiographic parameters were expected to be better in the robotic-assisted group.

## Material and methods

### Patients

This was a retrospective cohort study encompassing all patients who underwent medial BUKA performed either with robotic assistance or conventionally during a single surgical procedure from April 2016 to January 2022. All operations were performed by three high-volume UKA surgeons (>15 UKAs/year) in a single unit. Participants were excluded if they had another associated surgical procedure (anterior cruciate ligament reconstruction, patellofemoral prosthesis, osteotomy, total hip arthroplasty). In addition, any patient presenting with lower limb coronal plane deformity greater than 20° of varus, flexion <90°, and flexion contracture >10° was deemed unsuitable for UKA. In this study, candidates for UKA were not restricted by age, body mass index, or activity level. Eligible patients included those with an American Society of Anesthesiologists score of 3 or less who expressed a willingness to undergo a single-stage procedure. To ensure adequate postoperative assessment, a minimum follow-up period of 6 months was mandated.

### Data collection

Preoperatively, the following parameters were collected: age, gender, body mass index, and International Knee Society (IKS) score. Surgical time, defined as the duration from skin incision to wound closure, was recorded. The length of hospital stay was documented, considering outpatient surgery as a 0-day hospitalization. In addition, the need for blood transfusion was documented. Criteria for red blood cell transfusion included a hemoglobin level below 7.5 mg/dL or below 8 m/dL for patients with heart failure or symptomatic anemia (shortness of breath, dizziness, palpitations, or tachypnea). Postoperatively, at 2-month and at the last follow-up evaluation, patient-reported outcome measures (PROMs) were recorded using IKS score. Complications encountered within the first 60 days postoperatively were recorded and classified as major (death, pulmonary embolism, proximal deep-venous thrombosis, myocardial infarction, cardiac arrhythmia, and periprosthetic joint infection) or minor (superficial wound infection and distal deep vein thrombosis).

### Radiographic measurements

Radiographic parameters were calculated to evaluate implant positioning and alignment using a standardized radiographic protocol based on weight-bearing antero-posterior (AP) and lateral knee radiographs, patellar axial views, and full-length standing radiographs. Preoperative measurements included hip-knee-ankle angle (HKA) on long-leg radiographs and posterior tibial slope (PTS) in the medial compartment. Postoperative measurements were HKA angle, PTS, tibial component alignment relative to Cartier's angle (+: implant in varus; -: implant in valgus), and restoration of the height of the joint line (JL) (+: lower; -: higher JL) [[16], [17], [18], [19],^21^].

**Figure 1:**
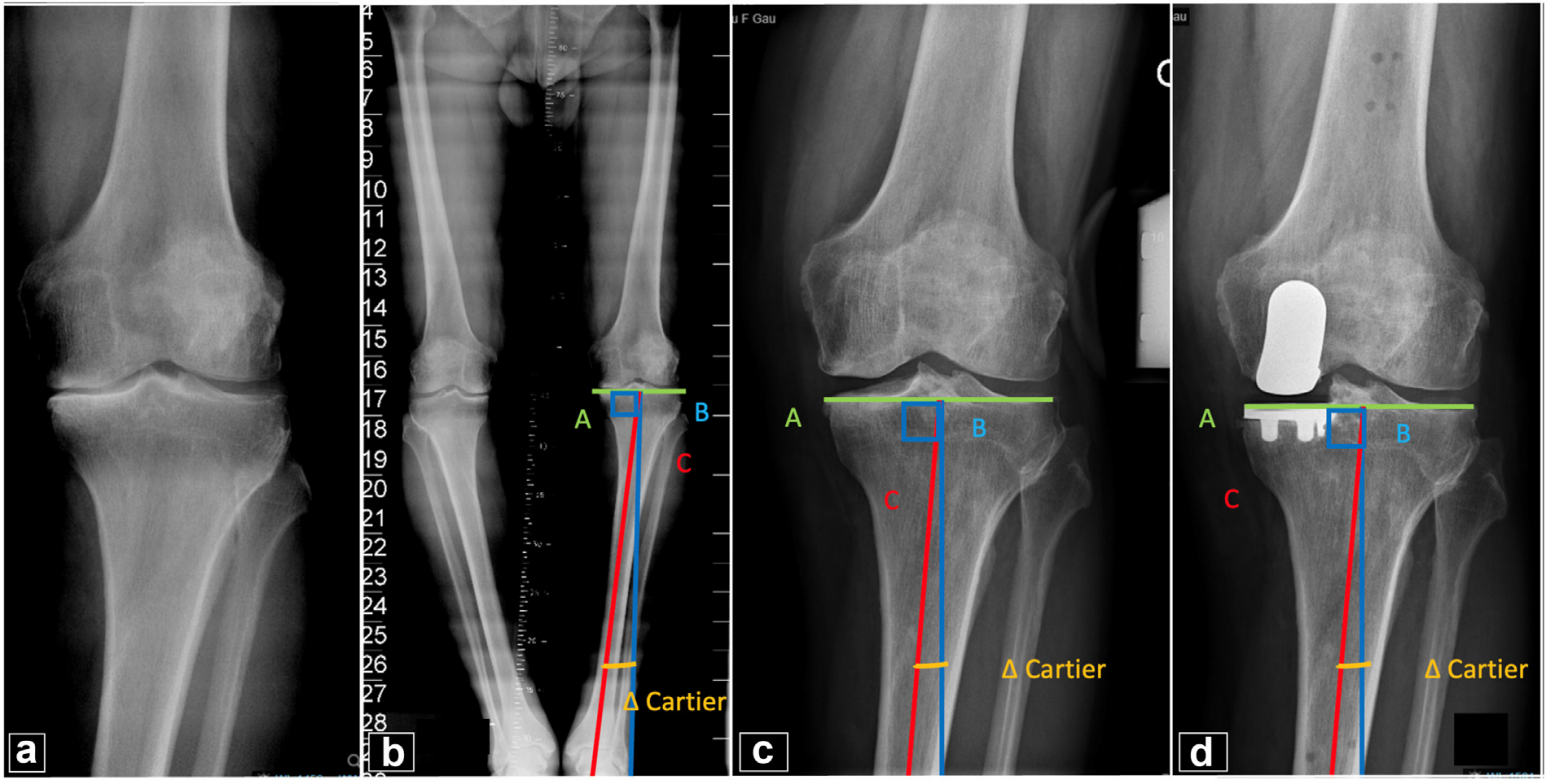
Cartier’s angle. (a) AP radiograph of the left knee showing medial femorotibial osteoarthritis. (b) Long leg x-ray showing how to measure the Cartier’ angle. Line A in green represents the tibial epiphyseal line. Line B, in blue, is perpendicular to line A. Line C, in red, represents the tibial mechanical axis. The angle formed between the line perpendicular to the tibial epiphyseal line (line B) and the mechanical axis of the tibia (line C) is the tibial epiphyseal angle, or Cartier's angle [22]. (c and d) Valgus stress radiograph and postoperative AP radiograph of the left knee showing how the implant has been aligned according to Cartier’s angle [21].

**Figure 2:**
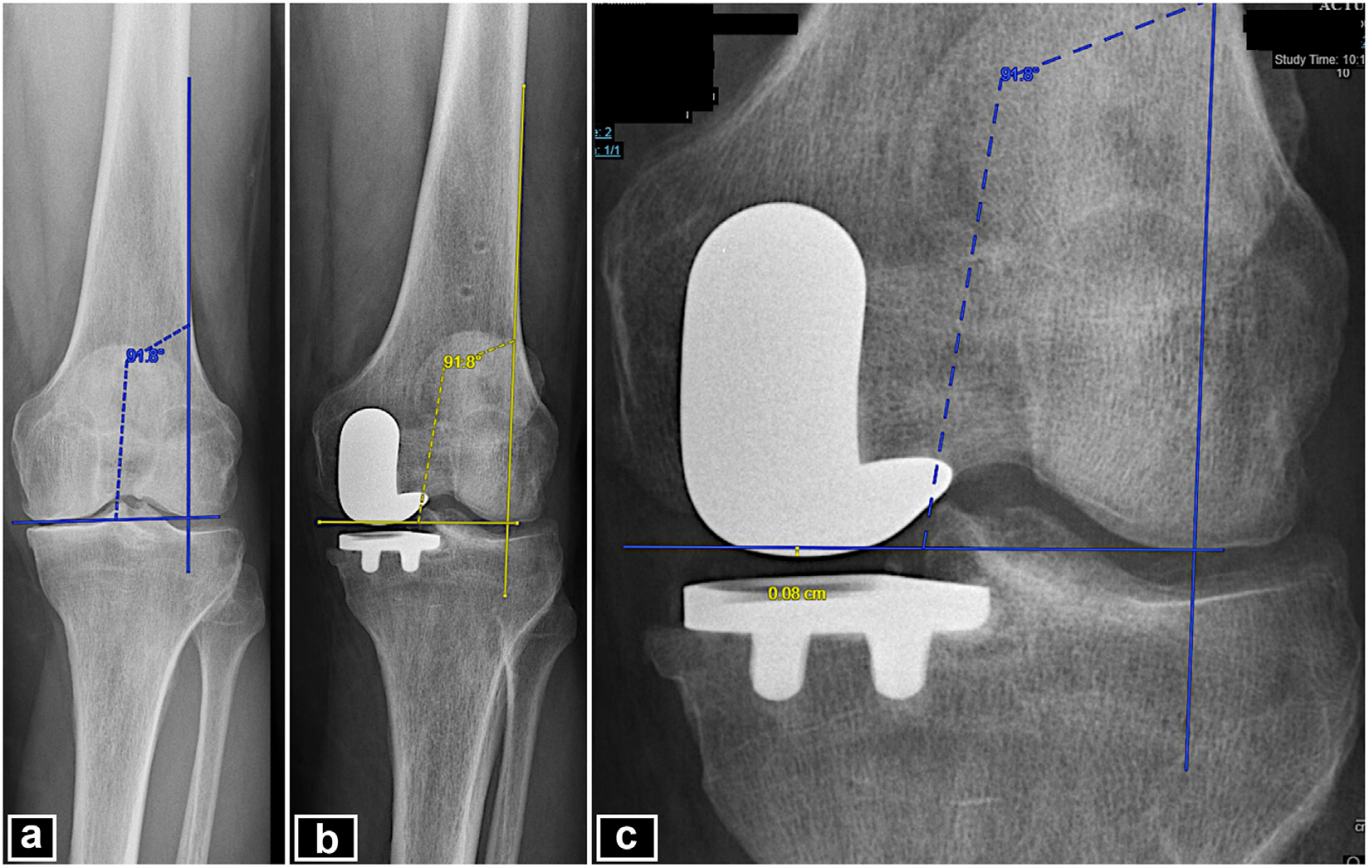
Preoperative and postoperative AP radiographs of left knee. (a) Measurement of joint line height using Herry’s method [17]. (b and c) Measurement of change in the height of the joint line using Herry’s method [17]. A joint line distalization of 0.8 mm can be observed. The joint line is measured by the angle formed between a line tangential to the lateral femoral cortical bone and another line that runs through the most distal point of the femoral condyles in a preoperative AP knee radiograph. On postoperative x-rays, the same angle measured preoperatively is applied to the lateral femoral cortex and to the distal end of the lateral femoral condyle. The distance between the most distal point of the femoral component and this line is measured in milliliters and is defined as the change in height of the joint line, as described and validated by Herry et al [18].

Cartier’s angle is formed by the proximal epiphysis and the tibial diaphyseal axis in an AP view of the knee. The alignment of the tibial component with Cartier's angle is measured by the angle formed between a line that runs tangential to the lateral tibial plateau and another line tangential to the tibial implant component [17,[19], [20], [21], [22]] (Fig. 1). The joint line height is measured by the angle formed between a line tangential to the lateral femoral cortical bone and another line that runs through the most distal point of the femoral condyles in a preoperative AP knee radiograph. On postoperative x-rays, the same angle measured preoperatively is applied to the lateral femoral cortex and to the distal end of the lateral femoral condyle. The distance between the most distal point of the femoral component and this line is measured in milliliters and is defined as the change in height of the JL, as described and validated by Herry et al [18,19] (Fig. 2).

All measurements were taken independently by 2 experienced knee surgeons, trained in UKA surgery, using Centricity Universal Viewer Zero Footprint software (6.0 SP77.0.2- GE Healthcare, Barrington, USA). The measurement accuracy was at one decimal. Outliers for postoperative measurements were defined as follows: HKA <175Ŷ° or >180°, PTS <2° or >8°, >3° or < -3° differences in Cartier’s angle, and ±2 mm changes in the height of the JL [23,24].

### Surgical protocol

The choice of surgical technique—conventional, image-based robotic (Mako, Stryker, Kalamazoo, MI, USA), or image-less robotic (BlueBelt Navio robotic surgical system, Smith & Nephew)—was determined based on system availability, and no predefined clinical indication for the use of a robotic system was employed. All of the patients operated with robotic assistance surgery before 2022 were done with the imageless system. After 2022, most of the UKA were done with an image-based system, and sometimes, depending on the number of surgeries per day and the surgeon’s preference, some patients were done with imageless system. Conventional procedures could be performed on both knees simultaneously by two teams or sequentially on each knee by the same team. Robotic procedures were only carried out by a single team in order not to interfere with the robot's performance.

All surgeries were performed using a mini-midvastus approach under spinal anesthesia. All three articular compartments and the cruciate ligaments were examined to confirm suitability for UKA. The tourniquet was inflated only during the cementation phase. Postoperative alignment targets were defined preoperatively, and they were: HKA between 175° and 180°, PTS between 2° and 8°, variations in Cartier’s angle less than 3°, and changes in the height of the JL less than 2 mm [23,24].

The implantation process involved cemented implants; Journey Uni implant (Smith & Nephew) was used for both conventional and image-less robotic surgeries (BlueBelt Navio robotic surgical system, Smith & Nephew), whereas the Restoris implant (Stryker) was utilized for surgeries conducted with the image-based robotic system (MAKO, Stryker Corp, Mako Surgical Corp, Ft. Lauderdale, Florida). Following surgery, a fast-track rehabilitation protocol [25] was implemented. Thromboprophylaxis was achieved with the administration of prophylactic dose enoxaparin for 30 days.

### Ethical approval

All procedures were performed in accordance with the ethical standards of the institutional and/or national research committee, the 1964 Helsinki Declaration and its later amendments, or comparable ethical standards. The Advisory Committee on Research Information Processing in the Field of Health approved this study in Paris on February 17, 2016, under number 16–140.

### Data analyses

A sample size calculation was performed based on the detection of a minimal clinically important difference on the IKS score postoperatively (=10 [26]). The estimated postoperative IKS of the conventional group was 90 with a standard deviation of 10. With beta and alpha = 5%, the number of subjects to be included was 52.

Mean value, standard deviation, minimum, and maximum values are presented for continuous variables. To evaluate normality in continuous variables, the skewness, kurtosis, and boxplots were observed, in addition to performing the Shapiro-Wilk and Kolmogorov-Smirnov tests. The Student’s t-test was used for independent variables that were normally distributed, and the paired Student’s t-test for paired, independent, and normally distributed variables. In cases where the distributional assumptions were not met, nonparametric tests were applied (Mann–Whitney U test for independent variables and the Wilcoxon signed-rank test for paired variables). Categorical variables are presented as absolute number, and percentages and differences among these were assessed using a chi-square test or Fisher’s exact test. The threshold for statistical significance was set at 5%. A post hoc power analysis was performed of each of the postoperative tested data in the result part. All analyses were performed using the XLSTAT software (version 2021, AddInsoft, Paris, France).

## Results

**Figure 3:**
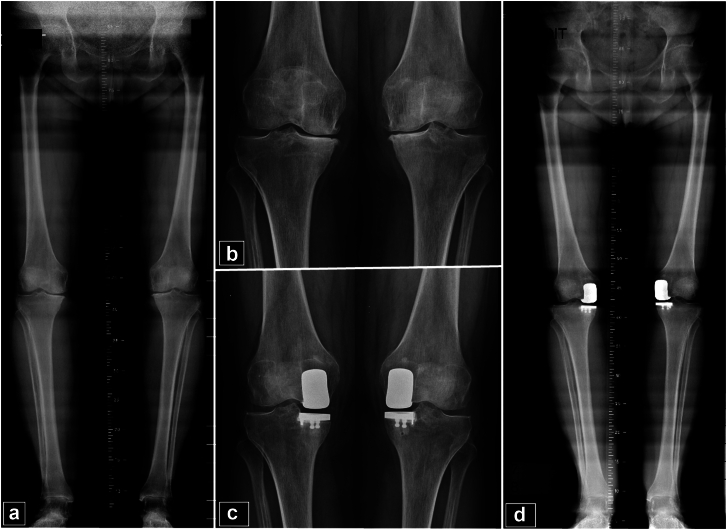
Bilateral medial unicompartmental knee arthroplasty. (a and b) Preoperative long-leg and AP views showing varus alignment and bilateral medial femorotibial knee osteoarthritis. (c and d) Postoperative AP and long-leg views showing bilateral medial unicompartmental knee arthroplasty after 12 months follow-up. The correct alignment of the prosthetic components is observed.

A total of 126 BUKAs were performed on 63 patients. Of these, 36 BUKAs (28.6%, 36/126) were conducted using a conventional technique, while the remaining 90 (71.4%, 90/126) utilized a robotic system. The mean follow-up duration was 21.9 ± 11 months [range 6.3-111.5]. In the robotic-assisted group, 26 BUKAs (28.9%) were done using image-based robotic assistance and 64 BUKAs (71.1%) using the imageless system. According to the BUKAs done with the conventional technique, 22 were done simultaneously (61.1%). A comparison of demographic and preoperative radiographic data revealed no significant differences between the conventional and robotic groups, as detailed in Table 1, Table 3. Out of the total procedures, 6 knees (4.7%, 6/126) had a follow-up period of less than 6 months and were consequently excluded from the postoperative analysis (Fig. 3).

**Table 1:** Demographic and preoperative data.

Demographic and preoperative data	Total (n = 126)	Conventional (n = 36)	Robotic (n = 90)	*P*-value
Women	33 (52.3%)	8 (44.4%)	25 (27.7%)	.16
Men	30 (47.7%)	10 (55.6%)	65 (72.3%)	
Age (y)	71.2 ± 6 [49-86]	71.2 ± 5.4 [54-85]	71.2 ± 6.1 [54-85]	.85
BMI (Kg/m2)	27.8 ± 3 [21–39]	28.6 ± 2.5 [23–33]	27.5 ± 2.8 [21–39]	.34
ASA	2 ± 0.3 [1–3]	2.1 ± 0.4 [1–3]	1.9 ± 0.4 [1–3]	.25
Preoperative IKS				
Knee (/100)	66.6 ± 8.9 [39-80]	64.25 ± 8.5 [39-77]	67.2 ± 8.9 [39-80]	.3
Function (/100)	72.3 ± 12.6 [0-90]	75 ± 8.3 [60-90]	71.7 ± 13.6 [0-90]	.9
Preoperative ROM (°)				
Extension	0.7 ± 1.3 [0-5]	0.8 ± 1.3 [0-5]	0.7 ± 1.3 [0-5]	
Fixed flexion contracture	0.5 ± 0.8 [0-5]	0.75 ± 1.2 [0-5]	0.43 ± 0.7 [0-5]	.3
Flexion	126.9 ± 5.4 [100-140]	126.9 ± 5.2 [110-140]	127 ± 5.4 [100-140]	.8

BMI, body mass index; ASA, American Society of Anesthesiologists; ROM, range of motion.

The duration of the robotic procedure was significantly longer than the conventional technique (115 ± 22 minutes vs 86.9 ± 12 minutes, *P* < .0001). Notably, no perioperative complications were recorded in either group, including the absence of complications related to the navigation pins. No patients required a transfusion. There was no significant difference in the length of hospital stay between the robotic and conventional groups (3.8 ± 1.4 days ± vs 3.2 ± 1.7, *P* = .10) (Table 2).

**Table 2:** Perioperative data, length of stay, clinical outcomes, and complications.

Demographic and preoperative data	Total (n = 120)	Conventional (n = 36)	Robotic (n = 84)	*P*-value	Post-hoc power
Follow- up (mo)	21.9 ± 11.6 [6.3-111.5]	22.25 ± 12.2 [7.2-102]	21.7 ± 11.4 [6.3-111.5]	.87	
Perioperative and stay data					
Time of surgery (min)	107 ± 21	86.9 ± 12	115 ± 22	<.0001	100%
Intraoperative complications	0	0	0	0	
Length of stay	3.7 ± 1.5 [1–13]	3.2 ± 1.7 [1–8]	3.8 ± 1.4 [1–13]	.10	46.2%
Patient-reported and clinical outcomes at 2 months					
IKS score					
IKS Knee (/100)	87.2 ± 7.5 [60-100]	85 ± 7.7 [60-100]	87.5 ± 6.6 [75-100]	.19	39.7%
IKS Function (/100)	86.25 ± 6.8 [60-100]	85 ± 8 [60-100]	86.9 ± 6.7 [75-100]	.30	23.9%
Range of motion (°)					
Extension	0.3 ± 0.6 [0-5]	0.5 ± 0.9 [0-5]	0.1 ± 0.2 [0-5]	**.04**	75.1%
Fixed flexion deformity	0.3 ± 0.6 [0-5]	0.7 ± 1.2 [0-5]	0 ± 1 [0-2]	**.02**	86.7%
Flexion	120 ± 6.2 [90-140]	119.1 ± 7.9 [100-140]	118.7 ± 7.8 [90-140]	.89	4.4%
Patient-reported and clinical outcomes at the last follow-up					
IKS score					
IKS Knee (/100)	90.9 ± 12.7 [30-100]	88.9 ± 14.3 [40-100]	91.7 ± 11.9 [30-100]	.36	17.7%
IKS Function (/100)	90.6 ± 13.7 [30-100]	87.5 ± 16.4 [30-100]	91.9 ± 11.9 [30-100]	.20	30.6%
Range of motion (°)					
Extension	0.3 ± 0.6 [0-5]	0.6 ± 1.1 [0-5]	0.2 ± 0.4 [0-5]	.08	56.5%
Fixed flexion deformity	0.2 ± 0.4 [0-10]	0.2 ± 0.5 [0-5]	0.1 ± 0.3 [0-10]	.61	20%
Flexion	124.9 ± 7.8 [90-145]	124.4 ± 6.6 [100-140]	124.1 ± 8.4 [90-145]	.67	4%
Early complications					
Etiology of complication				.99	
Blood transfusion	0	0	0		
Deep vein Thrombosis	0	0	0		
Pulmonary embolism	0	0	0		
Cardiovascular complication	0	0	0		
Stroke	0	0	0		
Fall	0	0	0		
Rehospitalization	0	0	0		
Wound complication	1 (0.8%)	0	1 (1.1%)		

Bold values indicate statistically significant data (*P* < .05).

At the 2-month follow-up, there were no significant differences in patient-reported outcomes between the robotic and conventional groups: IKS knee = 87.5 ± 6.6 vs 85 ± 7.7 (*P* = .19) and IKS function = 86.9 ± 6.7 vs 85 ± 8 (*P* = .3). These findings persisted at the last follow-up evaluation (21.9 ± 11 months, 6.3-111.5), IKS knee = 91.7 ± 11.9 vs 88.9 ± 14.3 (*P* = .36), IKS function = 91.9 ± 11.9 vs 87.5 ± 16.4 (*P* = .2) (Table 2).

The early complication rate was similar between the two groups (1.1% in the robotic arm vs 0% in the conventional arm, *P* = .99). No patients required readmission in any of the groups. The sole complication observed was wound drainage in the robotic group (not related to the navigation pins), which was successfully managed conservatively and no relapse was evident. We observed two revisions to TKA in the robotic group (2.2%), one due to tibial loosening after 10 months and another caused by anterior cruciate ligament mucoid degeneration after 15 months, both in the imageless robotic assisted group. In the conventional group, one revision was observed (2.8%) after 7 months caused by postoperative overcorrection and contralateral osteoarthritis progression.

With respect to radiological outcomes, implant positioning was more accurately achieved in the robotic-assisted group (varus alignment of the tibial implant 1° ± 1.8 vs 0.3 ± 0.6 [0-10], *P* = .03) as well as JL restoration (Δ = −1.4 ± 1.35 [−4 to 1] mm in conventional group vs −0.2 ± 0.7 [−2 to 2] mm in robotic group) (Table 3).

**Table 3:** Radiographic results.

Demographic and preoperative data	Total (n = 120)	Conventional (n = 36)	Robotic (n = 84)	*P*-value	Post-hoc power
HKA preoperative	173.5 ± 2.1 [168-179]	172.9 ± 2.1 [168-179]	173.8 ± 2.1 [167-178]	.13	
HKA postoperative	176.3 ± 2.2 [166-191]	176.4 ± 2.2 [168-185]	176.3 ± 2.3 [167-181]	1	4.1%
Outliers HKA	42 (35%)	14 (38.8 %)	28 (33.3%)	.53	
Slope preoperative	5.4 ± 2 [0-10]	5.5 ± 1.9 [1–9]	5.4 ± 2.1 [0-10]	.82	
Slope postoperative	3.4 ± 1.7 [0-13]	5 ± 1.8 [1–13]	2.7 ± 1.4 [0-8]	**<.0001**	100%
Outliers slope	33 (27.5%)	5 (13.8%)	8 (9.5%)	.48	
JL restitution (mm)	−0.5 ± 1 [-4 to 2]	−1.4 ± 1.4 [-4 to 1]	−0.2 ± 0.7 [-2 to 2]	**<.0001**	99.8%
Outliers JL restitution	0 (0%)	11 (30.6%)	0 (0%)	**<.0001**	
Δ Cartier	0.5 ± 1 [0-10]	1 ± 1.8 [0-10]	0.3 ± 0.6 [0-10]	**.03**	62.5%
Outliers Δ Cartier	10 (8.3%)	6 (16.6%)	4 (4.6%)	.06	

JL, joint line.

Bold values indicate statistically significant data (*P* < .05).

## Discussion

The most important finding of the present study was that conventional and robotic-assisted BUKA are associated with comparable early clinical outcomes and complications. Although the robotic approach had a longer operative time, this could be due to the possibility of performing the surgery simultaneously using conventional technique. On the other hand, radiographic parameters suggested improved implant positioning in the robotic-assisted group.

Robotic assistance was associated with a longer surgical time by 28 minutes; nevertheless, it remained within the range previously reported in the literature for conventional procedures (61-179 minutes, Table 4). The usual additional operating time caused by robotics is around 10 minutes [36]. The notably larger difference observed in our study can be primarily attributed to the ability to perform the conventional procedure with a double team, an approach not feasible with robotic operations due to potential interference with the sensors and availability of the robotic device. With respect to complications specific to the use of robotics, our findings of no procedure-related or pin-related complications align with the majority of the literature [[37], [38], [39]].

**Table 4:** Comparative results with the published international literature.

Demographic and preoperative data	N° of one-stage BUKA	Operative time (min)	Hospital stay (d)	Transfusion	Clinical outcomes	Complications
Chan et al. (2009) [27]	159	114	5	-	-	13 majors (8.2%)
Berend et al. (2011) [28]	70	109	1.7	0	IKS Function = 88	4 minors (5.7%)
Chen et al. (2013) [15]	248	130	5	1 (0.8%)	IKS Knee = 88 IKS function = 80 OKS = 17	3 majors (2.4%), 2 minors (1.6%)
Akhtar et al. (2014) [29]	76	83	3.5	0	Modified OKS = 34	1 major (1.3%), 2 minors (2.6%)
Winder et al. (2014) [30]	52	150	3.9	-	-	2 majors (3.8%)
Romagnoli et al. (2015) [1]	440	61.3	4	24 (10.9%)	-	9 majors (2%)
Ma et al. (2015) [31]	72	113.5	-	0	OKS = 18.3	1 major (1.4%), 2 minors (2.8%)
Ahn et al. (2017) [9]	104	-	8.4	9 (17.3%)	IKS knee 89.2 IKS function = 84.2	1 minor (0.9%)
Siedlecki et al. (2018) [32]	88	75.1	6.7	1 (2.3 %)	-	4 majors (4.5%), 2 minors (2.3%)
Clavé et al. (2018) [33]	100	-	-	3 (6%)	Modified OKS = 44.5 IKS = 192.6	3 majors (3%), 2 minors (2%)
Biazzo et al. (2019) [14]	102	93.2		4 (7.8%)	-	1 major (1%), 3 minors (2.9%)
Feng et al. (2019) [34]	78	120.2	4.2	1 (2.6%)	IKS knee = 88.9 IKS function = 80.9	4 minors (5.1%)
Sakka et al. (2020) [35]	238	147.1	1.1	0	-	2 majors (0.8%)
Gaggiotti et al. (2021) [11]	86	178.6	1.6	2 (4.7%)	IKS Knee = 80.9 IKS function = 89.8	6 minor (6.9%)
Actual serie	126	Robotic: 115 Conventional: 87	Robotic: 3.8 Conventional: 3.2	0	Robotic: -IKS Knee = 88.9 -IKS Function = 87.5 Conventional: -IKS Knee = 91.7 -IKS Function = 91.9	1 minor (0.8%)

OKS, Oxford Knee Score.

Patient-reported outcomes were comparable at 2 months, 6 months, and at the time of the last follow-up (21.9 ± 11 months, 6.3-111.5). Participants in the robotic group showed less residual fixed flexion deformity and superior extension at 2 months; however, this difference is unlikely to represent a meaningful clinical difference between the two groups. These results are in concordance with previous studies (Table 4) [40,41]. However, it is essential to consider that accurately identifying any early discrepancies in outcomes might require the analysis of more sensitive metrics, including opioid usage, duration until effective quadriceps recovery, and flexion restoration [42]. In addition, the PROMs employed in our research are validated tools for evaluating knee arthroplasty outcomes. Yet, it is key to acknowledge that the PROMs frequently used in research are subject to a notable ceiling effect, which may diminish their ability to distinguish between outcomes, particularly when patients achieve high scores [43].

In our study, there were minimal postoperative complications across both the robotic and conventional groups, with no patients requiring transfusion or any thromboembolic events reported. This favorable outcome likely reflects the rigorous patient selection process, which extends beyond the American Society of Anesthesiologists score to include considerations of the patient's activity level. In addition, adherence to anticoagulation guidelines with enoxaparin for 1 month postoperatively, a protocol not universally applied in studies reporting higher rates of thromboembolic events [27,44], may have contributed to these results. These findings reinforce the safety of the one-stage BUKA approach [11]. This is in line with conclusions from a recent systematic review that analyzed 10 articles, revealing that simultaneous BUKA offers comparable safety while associated with a reduced length of hospital stay and operative time [44].

Our research represents the first study to report on radiographic outcomes following BUKA utilizing robotic assistance. The primary advantage offered by the robotic system was the enhanced precision in implant placement, achieving superior control with respect to HKA, PTS, Cartier’s angle, and joint-line restoration. It has been shown that the precision of implant placement plays a critical role in the durability of UKA [16,24]. Key factors influencing outcomes include significant residual deformity, deviations in tibiofemoral alignment within both sagittal and coronal planes, and alterations in JL height [17,19,23,45].

This improved precision of robotic assistance has been previously documented [16,18,24], and while not directly correlating with improved clinical scores in our study, it could conceptually be associated with a reduced risk of revision in the medium term and high long-term survivorship [17,19,23,45,46]. However, an important consideration when employing robotics in BUKA is the potential for increased surgical time, as the use of robotic equipment precludes the possibility of performing simultaneous surgeries, even in a one-stage approach.

This study is subject to several limitations. Its retrospective design might have led to selection bias and restricted our capacity to gather more sensitive outcome measures. Such measures could have elucidated the superiority of one approach over the other concerning early recovery [42]. Also, as a nonrandomized retrospective study, the technique was chosen by the operator, and groups are not evenly divided. Additionally, our study did not encompass a healthcare economic evaluation that could have elucidated potential differences, especially in comparison with a control group undergoing two-stage operations. The sample size of our study may have resulted in limited statistical power, thus impeding our ability to identify significant differences, particularly concerning differences in survivorship and adverse events. Finally, surgeries within the robotic group utilized either an image-based or imageless robotic system, which could have introduced bias. Nonetheless, the use of cemented, fixed-bearing implants across both groups contributed to the homogeneity of the cohorts, mitigating potential discrepancies. The ramification of these results, particularly in relation to implantation accuracy, are yet to be realized, and further research is needed.

## Conclusions

This retrospective cohort study offers a comprehensive assessment of robotic-assisted medial BUKA, underscoring its safety and precision relative to the conventional technique. Although the robotic approach had a longer operative time, this could be due to the possibility of performing the surgery simultaneously using conventional technique. Clinical outcomes were comparable, and radiographic parameters suggested improved implant positioning in the robotic-assisted group. Future, longer-term studies encompassing a comprehensive range of PROMs and clinical outcomes are essential to fully study the utility of robotic assistance in the context of BUKA.

## CRediT authorship contribution statement

**Valentina Rossi:** Writing – original draft, Data curation. **Constant Foissey:** Writing – review & editing, Validation, Supervision, Methodology, Conceptualization. **Andreas Fontalis:** Writing – review & editing. **Gabriel Gaggiotti:** Writing – review & editing. **Stefano Gaggiotti:** Data curation. **Elvire Servien:** Supervision. **Sébastien Lustig:** Validation, Supervision, Conceptualization.

## Conflicts of interest

E. Servien receives institutional research support from Corin. S. Lustig is a consultant for Stryker, Smith Nephew, Heraeus, and Depuy Synthes; receives institutional research support from Corin, Lepine, and Amplitude; and is a deputy editor for JBJS (American), JEXO, and SICOT-j. All other authors declare no potential conflicts of interest.

For full disclosure statements refer to https://doi.org/10.1016/j.artd.2024.101594.

## Appendix A Supplementary data

Conflict of Interest Statement for Foissey

Conflict of Interest Statement for Fontalis

Conflict of Interest Statement for Gaggiotti

Conflict of Interest Statement for Lustig

Conflict of Interest Statement for Rossi

Conflict of Interest Statement for Servien

Conflict of Interest Statement for Gaggiotti

## References

[1] Romagnoli S., Zacchetti S., Perazzo P., Verde F., Banfi G., Viganò M. (2015). Onsets of complications and revisions are not increased after simultaneous bilateral unicompartmental knee arthroplasty in comparison with unilateral procedures. Int Orthop.

[2] Ledingham J., Regan M., Jones A., Doherty M. (1993). Radiographic patterns and associations of osteoarthritis of the knee in patients referred to hospital. Ann Rheum Dis.

[3] Sayeed S.A., Sayeed Y.A., Barnes S.A., Pagnano M.W., Trousdale R.T. (2011). The risk of subsequent joint arthroplasty after primary unilateral total knee arthroplasty, a 10-year study. J Arthroplasty.

[4] Newman J.H., Ackroyd C.E., Shah N.A. (1998). Unicompartmental or total knee replacement? Five-year results of a prospective, randomised trial of 102 osteoarthritic knees with unicompartmental arthritis. J Bone Joint Surg Br.

[5] Reilly K.A., Beard D.J., Barker K.L., Dodd C.A.F., Price A.J., Murray D.W. (2005). Efficacy of an accelerated recovery protocol for Oxford unicompartmental knee arthroplasty--a randomised controlled trial. Knee.

[6] Svärd U.C., Price A.J. (2001). Oxford medial unicompartmental knee arthroplasty. A survival analysis of an independent series. J Bone Joint Surg Br.

[7] Laurencin C.T., Zelicof S.B., Scott R.D., Ewald F.C. (1991). Unicompartmental versus total knee arthroplasty in the same patient. A comparative study. Clin Orthop Relat Res.

[8] Pandit H., Hamilton T.W., Jenkins C., Mellon S.J., Dodd C.A.F., Murray D.W. (2015). The clinical outcome of minimally invasive Phase 3 Oxford unicompartmental knee arthroplasty: a 15-year follow-up of 1000 UKAs. Bone Joint Lett J.

[9] Ahn J.H., Kang D.M., Choi K.J. (2017). Bilateral simultaneous unicompartmental knee arthroplasty versus unilateral total knee arthroplasty: a comparison of the amount of blood loss and transfusion, perioperative complications, hospital stay, and functional recovery. Orthop Traumatol Surg Res.

[10] Liddle A.D., Pandit H., Judge A., Murray D.W. (2015). Patient-reported outcomes after total and unicompartmental knee arthroplasty. Bone Joint Lett J.

[11] Gaggiotti G., Gaggiotti S., Gaggiotti S., Ringa J.C. (2023). Bilateral simultaneous unicompartmental knee arthroplasty. Medium-Term outcomes in 86 arthroplasties with an average follow-up of 6.2 years. Rev Asoc Argent Ortop Traumatol.

[12] Malahias M.-A., Gu A., Adriani M., Addona J.L., Alexiades M.M., Sculco P.K. (2019). Comparing the safety and outcome of simultaneous and staged bilateral total knee arthroplasty in contemporary practice: a systematic review of the literature. J Arthroplasty.

[13] Clavé A., Ros F., Letissier H., Flecher X., Argenson J.-N., Dubrana F. (2021). A case-control comparison of single-stage bilateral vs unilateral medial unicompartmental knee arthroplasty. J Arthroplasty.

[14] Biazzo A., Masia F., Verde F. (2019). Bilateral unicompartmental knee arthroplasty: one stage or two stages?. Musculoskelet Surg.

[15] Chen J.Y., Lo N.N., Jiang L., Chong H.C., Tay D.K.J., Chin P.L. (2013). Simultaneous versus staged bilateral unicompartmental knee replacement. Bone Joint Lett J.

[16] Foissey C., Batailler C., Vahabi A., Fontalis A., Servien E., Lustig S. (2023). Better accuracy and implant survival in medial imageless robotic-assisted unicompartmental knee arthroplasty compared to conventional unicompartmental knee arthroplasty: two- to eleven-year follow-up of three hundred fifty-six consecutive knees. Int Orthop.

[17] Foissey C., Batailler C., Vahabi A., Fontalis A., Servien E., Lustig S. (2023). Combination of a high residual varus and joint-line lowering strongly increases the risk of early implant failure in medial unicompartmental knee arthroplasty. J Arthroplasty.

[18] Herry Y., Batailler C., Lording T., Servien E., Neyret P., Lustig S. (2017). Improved joint-line restitution in unicompartmental knee arthroplasty using a robotic-assisted surgical technique. Int Orthop.

[19] Gaggiotti S., Foissey C., Pineda T., Batailler C., Gaggiotti G., Gaggiotti S. (2024). Enhancing robotic precision in medial UKA: image-based robot-assisted system had higher accuracy in implant positioning than imageless robot-assisted system across 292 knees. Knee Surg Sports Traumatol Arthrosc.

[20] Lee J.H., Jung H.J., Choi B.S., Ro D.H., Kim J.I. (2023). Effectiveness of robotic arm-assisted total knee arthroplasty on transfusion rate in staged bilateral surgery. J Clin Med.

[21] Gaggiotti S., Foissey C., Rossi V., Batailler C., Gaggiotti G., Gaggiotti S. (2024). Valgus stress knee radiographs accurately anticipate the bone resection in medial unicompartmental knee arthroplasty: protocol validation using an image-based robotic system. Knee Surg Sports Traumatol Arthrosc.

[22] Rivière C., Sivaloganathan S., Villet L., Cartier P., Lustig S., Vendittoli P.A. (2022). Kinematic alignment of medial UKA is safe: a systematic review. Knee Surg Sports Traumatol Arthrosc.

[23] Chatellard R., Sauleau V., Colmar M., Robert H., Raynaud G., Brilhault J. (2013). Medial unicompartmental knee arthroplasty: does tibial component position influence clinical outcomes and arthroplasty survival?. Orthop Traumatol Surg Res.

[24] Batailler C., White N., Ranaldi F.M., Neyret P., Servien E., Lustig S. (2019). Improved implant position and lower revision rate with robotic-assisted unicompartmental knee arthroplasty. Knee Surg Sports Traumatol Arthrosc.

[25] Gromov K., Petersen P.B., Jørgensen C.C., Troelsen A., Kehlet H. (2020). Lundbeck foundation centre for fast-track hip and knee replacement collaborative group. Unicompartmental knee arthroplasty undertaken using a fast-track protocol. Bone Joint Lett J.

[26] Lizaur-Utrilla A., Gonzalez-Parreño S., Martinez-Mendez D., Miralles-Muñoz F.A., Lopez-Prats F.A. (2020). Minimal clinically important differences and substantial clinical benefits for Knee Society Scores. Knee Surg Sports Traumatol Arthrosc.

[27] Chan W.C.W., Musonda P., Cooper A.S., Glasgow M.M.S., Donell S.T., Walton N.P. (2009). One-stage versus two-stage bilateral unicompartmental knee replacement: a comparison of immediate post-operative complications. J Bone Joint Surg Br.

[28] Berend K.R., Morris M.J., Skeels M.D., Lombardi A.V., Adams J.B. (2011). Perioperative complications of simultaneous versus staged unicompartmental knee arthroplasty. Clin Orthop Relat Res.

[29] Akhtar K.S.N., Somashekar N., Willis-Owen C.A., Houlihan-Burne D.G. (2014). Clinical outcomes of bilateral single-stage unicompartmental knee arthroplasty. Knee.

[30] Winder R.P., Severson E.P., Trousdale R.T., Pagnano M.W., Wood-Wentz C.M., Sierra R.J. (2014). No difference in 90-day complications between bilateral unicompartmental and total knee arthroplasty. Am J Orthop.

[31] Ma T., Tu Y.-H., Xue H.-M., Wen T., Cai M.-W. (2015). Clinical outcomes and risks of single-stage bilateral unicompartmental knee arthroplasty via Oxford phase III. Chin Med J.

[32] Siedlecki C., Beaufils P., Lemaire B., Pujol N. (2018). Complications and cost of single-stage vs. two-stage bilateral unicompartmental knee arthroplasty: a case-control study. Orthop Traumatol Surg Res.

[33] Clavé A., Gauthier E., Nagra N.S., Fazilleau F., Le Sant A., Dubrana F. (2018). Single-stage bilateral medial Oxford Unicompartmental Knee Arthroplasty: a case-control study of perioperative blood loss, complications and functional results. Orthop Traumatol Surg Res.

[34] Feng S., Yang Z., Sun J.-N., Zhu L., Wang S., Guo K.-J. (2019). Comparison of the therapeutic effect between the simultaneous and staged unicompartmental knee arthroplasty (UKA) for bilateral knee medial compartment arthritis. BMC Musculoskelet Disord.

[35] Sakka B.I., Shiinoki A., Morikawa L., Mathews K., Andrews S., Nakasone C. (2020). Comparison of early post-operative complications following unilateral or single-stage bilateral unicompartmental knee arthroplasty. Knee.

[36] Mancino F., Cacciola G., Malahias M.-A., De Filippis R., De Marco D., Di Matteo V. (2020). What are the benefits of robotic-assisted total knee arthroplasty over conventional manual total knee arthroplasty? A systematic review of comparative studies. Orthop Rev.

[37] Mergenthaler G., Batailler C., Lording T., Servien E., Lustig S. (2021). Is robotic-assisted unicompartmental knee arthroplasty a safe procedure? A case control study. Knee Surg Sports Traumatol Arthrosc.

[38] Lonner J.H., Kerr G.J. (2019). Low rate of iatrogenic complications during unicompartmental knee arthroplasty with two semiautonomous robotic systems. Knee.

[39] Blyth M.J.G., Anthony I., Rowe P., Banger M.S., MacLean A., Jones B. (2017). Robotic arm-assisted versus conventional unicompartmental knee arthroplasty: exploratory secondary analysis of a randomised controlled trial. Bone Joint Res.

[40] Gaudiani M.A., Samuel L.T., Kamath A.F., Courtney P.M., Lee G.-C. (2021). Robotic-assisted versus manual unicompartmental knee arthroplasty: contemporary systematic review and meta-analysis of early functional outcomes. J Knee Surg.

[41] Zhang J., Ng N., Scott C.E.H., Blyth M.J.G., Haddad F.S., Macpherson G.J. (2022). Robotic arm-assisted versus manual unicompartmental knee arthroplasty : a systematic review and meta-analysis of the MAKO robotic system. Bone Joint Lett J.

[42] Kayani B., Konan S., Tahmassebi J., Rowan F.E., Haddad F.S. (2019). An assessment of early functional rehabilitation and hospital discharge in conventional versus robotic-arm assisted unicompartmental knee arthroplasty. Bone Joint Lett J.

[43] Fontalis A., Kayani B., Haddad I.C., Donovan C., Tahmassebi J., Haddad F.S. (2023). Patient-reported outcome measures in conventional total hip arthroplasty versus robotic-arm assisted arthroplasty: a prospective cohort study with minimum 3 Years’ follow-up. J Arthroplasty.

[44] Malahias M.-A., Manolopoulos P.P., Mancino F., Jang S.J., Gu A., Giotis D. (2021). Safety and outcome of simultaneous bilateral unicompartmental knee arthroplasty: a systematic review. J Orthop.

[45] Kazarian G.S., Barrack T.N., Okafor L., Barrack R.L., Nunley R.M., Lawrie C.M. (2020). High prevalence of radiographic outliers and revisions with unicompartmental knee arthroplasty. J Bone Joint Surg Am.

[46] Bayoumi T., Kleeblad L.J., Borus T.A., Coon T.M., Dounchis J., Nguyen J.T. (2023). Ten-year survivorship and patient satisfaction following robotic-arm-assisted medial unicompartmental knee arthroplasty. J Bone Joint Surg Am.

