# Supplementary material for: Robotic Assistance in Simultaneous Bilateral Medial Unicompartmental Knee Arthroplasty: A Retrospective Cohort Study of 126 Knees Demonstrating Enhanced Radiographic Accuracy and Comparable Safety to Conventional Methods
Source: Arthroplast Today. 2025 Jan 21;31:101594. doi: 10.1016/j.artd.2024.101594 (PMC11788786; doi:10.1016/j.artd.2024.101594)
Supplement: Conflict of Interest Statement for Fontalis [file mmc2.docx]

# INDIVIDUAL CONFLICT OF INTEREST STATEMENT

***The Journal of Arthroplasty***

(Adopted from the American Academy of Orthopaedic Surgeons disclosure statement)

The following form **must be filled out completely and submitted by each author (example, 6 authors, 6 forms).**

**All items require a response. If there is no relevant disclosure for a given item, enter "*None*.”**

**Robotic-arm Assistance in Simultaneous Bilateral Medial Unicompartmental Knee Arthroplasty:** **A** **Retrospective Cohort Study of 126 Knees Demonstrating Enhanced Radiographic Accuracy and Comparable Safety to Conventional Methods.**

1. Royalties from a company or supplier (The following conflicts were disclosed)

None

2. Speakers bureau/paid presentations for a company or supplier (The following conflicts were disclosed)

None

3A. Paid employee for a company or supplier (The following conflicts were disclosed)

None

3B. Paid consultant for a company or supplier (The following conflicts were disclosed)

None

3C. Unpaid consultants for a company or supplier (The following conflicts were disclosed)

None

4. Stock or stock options in a company or supplier (The following conflicts were disclosed)

None

5. Research support from a company or supplier as a Principal Investigator (The following conflicts were disclosed)

None

6. Other financial or material support from a company or supplier (The following conflicts were disclosed)

None

7. Royalties, financial or material support from publishers (The following conflicts were disclosed)

None

8. Medical/Orthopaedic publications editorial/governing board (The following conflicts were disclosed)

None

9. Board member/committee appointments for a society (The following conflicts were disclosed)

None

**Each author must sign AND print or type his/her name, date and submit a separate form**


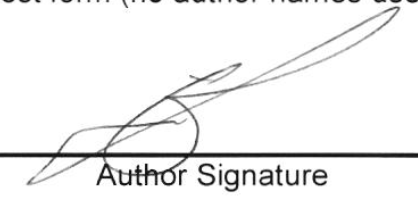
In addition, one BLINDED Conflict of Interest form (no author names used) should be submitted per manuscript with all author disclosures.

Andreas Fontalis 12/03/2024

Author Name (Print or Type) Author Signature Date
